# The Role of Post-Translational Modifications on Prion-Like Aggregation and Liquid-Phase Separation of FUS

**DOI:** 10.3390/ijms19030886

**Published:** 2018-03-16

**Authors:** Shannon N. Rhoads, Zachary T. Monahan, Debra S. Yee, Frank P. Shewmaker

**Affiliations:** Department of Pharmacology and Molecular Therapeutics, Uniformed Services University of the Health Sciences, Bethesda, MD 20814, USA; shannon.rhoads.ctr@usuhs.edu (S.N.R.); zachary.monahan@usuhs.edu (Z.T.M.); debra.yee.ctr@usuhs.edu (D.S.Y.)

**Keywords:** FUS, ALS, FTLD, prion, amyloid, LLPS

## Abstract

Subcellular mislocalization and aggregation of the human FUS protein occurs in neurons of patients with subtypes of amyotrophic lateral sclerosis and frontotemporal dementia. FUS is one of several RNA-binding proteins that can functionally self-associate into distinct liquid-phase droplet structures. It is postulated that aberrant interactions within the dense phase-separated state can potentiate FUS’s transition into solid prion-like aggregates that cause disease. FUS is post-translationally modified at numerous positions, which affect both its localization and aggregation propensity. These modifications may influence FUS-linked pathology and serve as therapeutic targets.

## 1. The Link between FUS and Neurodegenerative Disease

FUS (fused in sarcoma) gets its name from forming oncogenic fusion proteins with specific transcription factors following chromosomal rearrangements [1]. Such rearrangement is common in liposarcomas, thus FUS also goes by the name TLS (translocated in liposarcoma). Since 2009, FUS has received more attention for its connection to neurodegenerative disease after missense mutations were discovered to cause a small percentage of cases of amyotrophic lateral sclerosis (ALS-FUS) [2,3]. In the motor neurons of these patients, the normally nuclear FUS protein was found in cytoplasmic proteinaceous inclusions. Since then, non-mutant FUS has been identified in cytoplasmic inclusions in cortical neurons of a subset of patients with frontotemporal dementia (FTD; the neuropathological diagnosis is termed frontotemporal lobar degeneration (FTLD-FUS)) [4]. Both ALS and FTD are incurable and their clinical and pathological overlap suggests that they are part of a disease continuum.

Mutations in FUS are autosomal dominant causes of familial ALS. Most mutations alter the C-terminal nuclear localization signal, resulting in excess cytoplasmic FUS that can form inclusions with gain-of-function toxicity [5]. Whether ALS-FUS or FTLD-FUS, it is the accumulation of FUS into cytoplasmic aggregates that appears to cause neuronal loss. The clinical presentation likely depends on the specific type of neurons that are affected. Biophysical and histological analysis suggest that FUS cytoplasmic aggregation may spread across anatomical networks through a prion-like mechanism [6]. Therefore, future drugs may target FUS’s ability to cytoplasmically localize and/or form proteinaceous aggregates. Extensive post-translational methylation and phosphorylation of FUS have been shown to influence localization and aggregation, respectively. Here we review the post-translational modifications (PTMs) of FUS in the context of how they affect function, self-association and pathology. 

## 2. FUS Structure and Function

**Figure 1:**
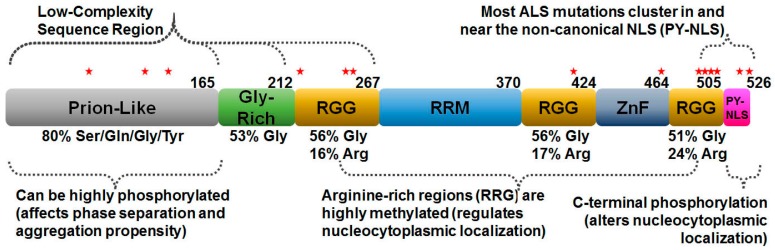
Schematic of human FUS domain organization. Approximately the first half of FUS has little sequence complexity and consists mostly of a few different amino acids. This region is sometimes called the low-complexity domain. The prion-like domain (PrLD) shares sequence composition with domains in yeast proteins that form self-replicating amyloid structures (i.e., prions). FUS’s PrLD is highly phosphorylated following certain stresses. The RGG domains contain the triplet repeat motif of arginine–glycine–glycine, which are extensively methylated. FUS also contains an RNA-recognition motif (RRM), a zinc-finger domain (ZnF), and a proline–tyrosine nuclear localization signal (PY–NLS). The red stars indicate ALS mutation sites that are also post-translationally modified (see Table 1).

FUS is a ubiquitously expressed, predominantly nuclear, metazoan protein. Many different functions—primarily involving RNA metabolism and processing—have been ascribed to FUS. Its domain architecture is presented in Figure 1. The amino-terminal prion-like domain (PrLD) has garnered much attention because its composition is similar to the domains found in yeast proteins that form self-propagating amyloid fibers (described below). FUS also contains multiple arginine/glycine-rich regions (RGG; named for a repeated arginine–glycine–glycine motif), an RNA-recognition motif (RRM), zinc finger (ZnF), and nuclear export (NES) and localization sequences (NLS).

The physiological functions of FUS are not fully characterized and remain an area of active investigation. Early work with FUS-knockout organisms suggested a critical developmental function, but specific molecular activities remained ambiguous [7,8,9,10]. One of the most challenging aspects of understanding FUS physiology is its apparent diversity of functions. Broadly, these functions can be divided into three categories: DNA damage response, RNA metabolism, and the cellular stress response [11,12,13,14,15,16,17]. That it adopts such diverse roles suggests an accessory role for FUS in each of these categories; were it a central player in any category, we may expect its physiological repertoire to be more limited to that function.

In the DNA damage response (DDR), FUS has been shown to localize at sites of laser-induced DNA damage [18,19], to interact with HDAC1-mediated DNA repair pathways [20], and to interact with poly-ADP ribose, a by-product of DNA damage [18,19]. Disease-associated mutations limit FUS’s participation in DDR [21], perhaps due to enhanced cytoplasmic mislocalization [22]. Moreover, studies have now also shown post-translational modification of FUS to be coincident with DNA damage [18,23]. This suggests that PTMs are a possible modifier of its function in these pathways. Most telling, studies have documented increased evidence of DNA damage in cells expressing mutant FUS [20]. Therefore, while its precise function is still unclear, the function of FUS in the DDR is clearly consequential.

The most well-established roles for FUS in RNA metabolism involve three critical functions: mRNA transport and translation [13,14], gene splicing [11,12,20,24], and gene expression [17,25,26]. Each of these functions involves association of FUS with other players, including molecular motor proteins such as Myo5A and KIF5B for mRNA transport [8,27,28], and U11 snRNP for splicing functions [20,29]. Additionally, FUS has been shown to interact with transcriptional regulators, RNA pol II, and regulatory regions of DNA, thus mediating mRNA synthesis [8,12,17,25,26]. Disease-associated mutations in FUS have been shown to impair its role in many of these functions [30,31].

The response of FUS to general cellular stress involves association with ribonucleoprotein (RNP) bodies/granules—which are transient regulatory structures. Examples include stress granules (SGs) and processing bodies (P-bodies), which are sites of altered RNA metabolism, in response to specific stressors. The formation of FUS-positive SGs and P-bodies has been observed in the face of osmotic stress, as well as heat-shock [8,16,32]. FUS’s roles in RNA processing functions may explain its association with these structures [16].

The three general functions for FUS listed above are disparate from one another. Nevertheless, one unifying principle is that FUS must have a mode of protein–protein interaction that facilitates localization at different subcellular sites that perform different functions. This ability to localize at DNA lesions, with other RNA processing proteins at mRNAs, and at sites of SG formation may explain why FUS has retained such a robust capacity for self-association and liquid-like phase separation (discussed below).

### 2.1. FUS Can Undergo Liquid–Liquid Phase Separation

Some biochemical reactions are spatially and temporally localized into discrete subcellular microenvironments that lack separating membranes. This can be achieved through liquid–liquid phase separation (LLPS), a process by which macromolecules separate from the bulk solvent into a distinct liquid phase [77]. These droplet-like microenvironments flow and fuse like liquids and tend to adopt spherical shapes [78,79]. They can assemble and disassemble in seconds, providing cells with the ability to respond to signals rapidly and specifically. Types of phase-separated structures include nucleoli [80], P-bodies [81], SGs [82], Cajal bodies [83], and sites of DNA damage [84]. Human FUS is hypothesized to undergo LLPS during inclusion in RNP bodies, mediating one of its many normal cellular functions discussed above.

Common features among proteins capable of undergoing LLPS include: long intrinsically disordered domains; domains with repeated motifs; and modular molecular-interaction domains (e.g., RNA-recognition motifs (RRMs)) [77,85]. Long intrinsically disordered domains generally have low sequence complexity and form complex structural ensembles that do not hydrophobically collapse. These domains and repeated sequence motifs appear to support the network of dynamic interactions enabling a phase-separated state without incurring a strong entropic penalty. FUS’s amino-terminal PrLD is intrinsically disordered, contains multiple repeats of a S/GYS/G motif and is critical to FUS’s condensation into a droplet state [12]. The repeat motif has the potential to be dramatically altered through tyrosine and serine phosphorylation. Electrostatic interactions between macromolecules are a known driving force for many systems that undergo LLPS [86], thus adding numerous negatively charged phosphates to the PrLD repeats is theoretically a means to regulate FUS’s LLPS within cells (discussed further below) [87].

FUS’s RRM is also likely involved in cellular LLPS. In vitro, recombinant FUS’s phase separation is enhanced by the addition of RNA [12]. For many RNA-binding proteins that undergo LLPS, the addition of RNA generally decreases the saturation concentration for phase separation [77]. For example, the interaction between RNA and the RRM domains of hnRNPA1—a protein with many similarities to FUS—lowers the critical concentration for phase separation [82]. This may result from RNA’s capacity for multivalent binding with numerous RRM domains enabling a network of interactions in the phase-separated state. PTMs that alter RRM binding would thus be a means to regulate LLPS. A recent bioinformatic study found that human RRM domains are among the most heavily modified domains in the proteome [88]. Interestingly, tyrosines within RNA-binding sites are commonly phosphorylated. Tyrosines 304 and 325 in the FUS RRM are both predicted phosphorylation sites in silico, and by mass spectrometry (Table 1), suggesting that FUS RRM-RNA interactions that mediate LLPS may be under the control of tyrosine kinases. The RRM and PrLD domains have received the most focus, but other domains and their PTMs may also contribute to LLPS. For example, removal of FUS’s RRM is not sufficient to completely abrogate its RNA binding, so PTMs of other domains could also influence multivalent RNA interactions in the phase-separated state [14].

### 2.2. FUS Can Form Prion-Like Solid Aggregates

A distinguishing characteristic of most neurodegenerative diseases is that a single disease-linked protein aggregates in specific neuronal cells or tissue. In ALS, this happens in motor neurons; the specific pathological protein may be FUS, TDP-43, SOD1 or one of several other proteins [89]. Patient pathology follows not from aggregation occurring in an isolated cell, but through entire neural networks [6]. This pattern of pathology suggests propagation through physical contact, much like prion protein (PrP), whose pathological conformation spreads through tissue in the fatal transmissible spongiform encephalopathies. Importantly, the misfolded form of PrP is itself toxic and serves as a template for other PrP molecules to convert into the pathological form [90]. Similarly, the aggregated form of FUS demonstrates gain-of-function toxicity in multiple cell and animal models [5,91,92,93]. ALS-linked FUS mutations have been shown to increase FUS’s propensity to form solid aggregates [84,94]).

A structural mechanism at the molecular level must underlie the faithful, yet catastrophic, propagation of specific protein aggregates. The single most unifying molecular model for misfolded protein propagation is amyloid, which is a filamentous protein homopolymer, with a high degree of structural order at the atomic level (i.e., amyloid is folded, albeit misfolded, but not structurally disorganized). Many different types of proteins can form amyloid, but much like a one-dimensional crystal, any specific amyloid filament is composed of repeated protein units of identical (or near identical) amino acid sequence. Protein backbones are aligned perpendicularly to the amyloid fiber axis in a configuration called “cross-β”. The most common amyloid architecture has each polypeptide aligned in-register with the next polypeptide forming parallel β sheets that run the length of the fiber axis. All yeast prion domains—from which FUS’s amino-terminal domain gets its name—form parallel in-register amyloid in their infectious forms [95].

An amyloid configuration, based on parallel in-register β sheets, depends on each amino acid lying adjacent to its exact counterpart in the next polypeptide. Introducing repulsive charged groups or other PTMs is structurally disruptive to this crystal-like arrangement. It is for this reason that PTMs, specifically within an amyloid-forming domain, may play an important role in disrupting FUS’s pathological aggregation. Using solid-state NMR, Murray and coworkers identified a core region of FUS’s PrLD (amino acids 39–95) that forms amyloid with parallel in-register β sheet structure [35]. Importantly, this region overlaps with multiple putative sites of phosphorylation (Table 1) [33]; this is predicted to have strong influence on FUS’s capacity to form solid aggregates (discussed below).

While FUS is demonstrably prone to aggregation [91,96], the architecture of its aggregate in diseased neurons is undetermined. When stained with thioflavin T dye, pathological FUS-positive inclusions do not yield a strong fluorescence like most amyloid-forming proteins [91]. However, this may be a peculiarity unique to FUS aggregates given that the PrLD of FUS unambiguously forms amyloid, while still exhibiting weak fluorescence responses to thioflavin T ([35] and unpublished observations). In summary, the most parsimonious explanation for FUS pathology is that it is based on prion-like, self-propagating, toxic aggregates for these reasons: FUS is autosomal dominant and neuronal cytoplasmic inclusions have gain-of-function toxicity; FUS inclusions follow distinct anatomical pathways in FUS-linked FTLD; FUS’s PrLD forms archetypical amyloid providing a structural mechanism for self-propagation.

### 2.3. FUS-Linked Disease May Result from an Irreversible Liquid-to-Solid State Transition 

Regardless of the atomic arrangement of FUS within neuronal inclusions, the phase-separated state has been implicated in potentiating the conversion into the solid-like pathological form [84]. Specifically, the high concentration of FUS in RNP bodies could stochastically facilitate an irreversible liquid-to-solid phase transition, especially under conditions involving mutant FUS and/or persistent RNP granules [97]. This hypothesis partly stems from the observation that many RNP body-associated proteins with prion-like domains are found in solid inclusions in neurodegenerative diseases [98], and from observations that prion-like domains appear to facilitate both phase separation and solid aggregate formation [82].

In vitro, FUS maintains a broad dynamic structural ensemble within liquid droplets; transient weak interactions, not solid-state interactions, facilitate phase separation [12]. However, if FUS droplets are subjected to multiple rounds of melting and re-separating, eventually they lose their pliability and resist melting [99]. These intractable droplets may form a ′glassy solid′ in which liquid-like unstructured conformations become relatively locked [77], or they may form a ′hydrogel′ composed of structured amyloid polymers [35]. Additionally, dense solid aggregates have been observed projecting from “locked” droplets, suggesting that interactions within the droplet state give rise to a solid-state conformation [84]. Disease-associated mutations that increase cytoplasmic localization of FUS can subsequently lead it to have more persistent association with cytoplasmic RNP bodies [100]. Also, mutant FUS is more prone to form a “locked” droplet state [99]. Thus, any PTM that affects FUS’s phase separation or inclusion in RNP bodies may likewise affect FUS’s potential to form pathological aggregates (discussed below). However, the fidelity with which these in vitro observations translate to FUS behavior in vivo is unclear.

## 3. Post-Translational Modification of FUS

PTMs have been suspected as critical mediators for pathological protein aggregation for years, especially in the context of neurodegenerative disease. For example, hyperphosphorylation of tau is widely suspected to be important in the pathogenesis of tauopathies, possibly by favoring the dissociation of tau from microtubules and thus favoring self-association and pathological aggregation [101]. However, others hypothesize that hyperphosphorylation disfavors tau aggregation, and thus may act as a protective mechanism [102,103]. Similarly opposing models for the role of phosphorylation in regulating protein self-assembly have emerged for other pathogenic proteins, such as alpha synuclein [104,105], TDP-43 [106,107], and FUS (described below). Thus, the precise roles of PTMs in facilitating pathology are not known. Likewise, the roles of PTMs in FUS pathology remain unresolved, despite observations that FUS undergoes extensive and complicated modification. Many of the sites that are modified are also known ALS mutation sites (Table 1, gray highlights). The most studied PTMs of FUS are serine/threonine phosphorylation and arginine methylation (Table 1).

### 3.1. FUS Phosphorylation

Prion-like amino acid sequences, being enriched in the residues serine, threonine, glutamine, asparagine, and tyrosine, have a high potential for phosphorylation. Not surprisingly, FUS phosphorylation has been extensively demonstrated in FUS’s PrLD (Figure 1), as well as other domains (Table 1). Among the earliest work, FUS proteolysis was shown to be regulated by Ser-256 phosphorylation in its first RGG domain [52] (Figure 1). Later, more abundant phosphorylation was observed within the PrLD, described above as the mediator of FUS self-assembly and phase separation. Several studies have detected phosphorylation within this region using mass spectrometry, producing 32 putative sites at either serine or threonine residues (Table 1). No tyrosine phosphorylation in the PrLD has been detected in this manner—possibly due to technical challenges—despite being abundant in the repeated S/GYS/G motifs. Though threonine is not part of the repeated motif, multiple phospho-threonines have been identified.

Of the identified candidate phosphorylation sites in the PrLD, the first to be confirmed in mammalian cells by alternative methods was serine 42 using a site-specific antibody [23]. The responsible kinase was proposed to be ATM (ataxia-telangiectasia mutated ser/thr kinase). Gardiner and colleagues suggested that phosphorylation modulated the response of FUS to DNA damage but did not describe the specific role of phosphorylated FUS. Later, Deng and colleagues examined the effects of phosphorylation on FUS [18]. They characterized FUS phosphorylation in the PrLD and determined that it follows DNA-damage. They concluded that phosphorylation is mediated by DNA-PK at S/TQ motifs in the PrLD, and hypothesized that this could facilitate FUS cytoplasmic localization. Broadly, these findings were consistent with the results of two independent groups who demonstrated that FUS accumulation follows DNA damage at the site of lesions, and its assembly there is dependent on its PrLD [15,21,108]. Importantly, accumulation at lesion sites was not dependent on phosphorylation. Taken together, a model emerged wherein FUS phosphorylation mediated its subcellular localization immediately following its intranuclear role in the DNA damage response. Understanding the precise consequences of phosphorylation is critical given that cytoplasmic accumulation is considered one of the potentiators of disease.

In our recent findings, DNA-PK-dependent phosphorylation appeared to decrease the aggregation propensity of FUS [36]. Assays using yeast, human cell lines, and recombinant proteins established that both phase separation and toxic FUS aggregation are retarded by phosphorylation and/or phosphomimetic substitution in the PrLD [35,36]. Both phosphorylation and its mimetic quantitatively reduce the prion-like nature of the PrLD by introducing electrostatic charges [36], thus offering an explanation for how these modifications reduce the propensity of the FUS PrLD to adopt the archetypal amyloid conformation and form cytoplasmic inclusions [35,109].

Separately, Lin and colleagues exploited a phase-separating in vitro model composed of poly-Src homology 3 (SH3) domain protein and poly-proline–rich-motif ligand to establish the effects of phosphorylation on phase separation [110]. When the FUS PrLD was fused to SH3, the system phase separated at lower concentration. Treating the system with DNA-PK to phosphorylate FUS PrLD eliminated the phase-separated droplets. All of these recent efforts are consistent with earlier work demonstrating that FUS PrLD retention within a hydrogel is disrupted by DNA-PK-dependent phosphorylation [38], but offer revelations for exactly how phosphorylation may regulate PrLD self-association [35], as well the consequences of phosphorylation on pathological aggregation, cytotoxicity, and physiological phase separation [36,110]. 

These results are also consistent with a broader biophysical phenomenon which is now emerging regarding the relationship of phosphorylation and protein-self association into phase-separated states. In a recent model system using cationic peptides and RNA, phosphorylation dramatically suppressed molecular condensation. Introduction of a single phosphoserine was sufficient to generate this effect [86]. The complex and extensive phosphorylation of FUS’s PrLD suggests that phosphorylation is capable of regulating FUS’s inclusion into RNP bodies. However, regulation of phase separation may not be limited to PrLD phosphorylation. Recent work showed that phosphorylation of S48 in TDP-43, distant from its own prion-like domain, altered phase separation in a recombinant protein system [111].

While critical to our understanding of FUS-linked pathology, the direct consequences of PrLD phosphorylation on FUS self-assembly does not address the finding previously suggested by Deng and colleagues. They suggested that phosphorylation played a role in mediating sub-cellular localization. We recently characterized conditions in which FUS PrLD phosphorylation is induced in human cells [33]. Phosphorylation at DNA-PK consensus sites—which were confirmed by antibodies specific to phosphoserines 26 and 30—did not directly alter subcellular localization of FUS. We additionally found that phosphorylation within the PrLD is extensive under some conditions, but occurs at lower frequencies under others, suggesting that differential phosphorylation may result in finer regulation of FUS activity.

While N-terminal phosphorylation seems to mediate self-association and not localization, C-terminal phosphorylation within the NLS (Figure 1) has been reported to disrupt the binding of nuclear import machinery, resulting in a higher cytoplasmic concentration of FUS [76,112]. One study found that the final amino acid, tyrosine 526, is phosphorylated by a Src family kinase [76]. Phosphorylation at this point decreased the binding affinity between FUS and transportin-1 (also known as Karyopherin β2), the nuclear import receptor of FUS responsible for shuttling FUS through the nuclear pore. Ultimately, this led to decreased nuclear import and accumulation of FUS in the cytoplasm. Additionally, another study found that phosphomimetic substitution at a different site within the NLS, serine 513, exacerbated the cytoplasmic tendency of mutant FUS constructs [112]. The mimetic by itself had no effect on localization, but presumably was affecting transportin-1 binding when coupled with a disease-associated NLS mutation. Though these results were done using an artificial construct, S513 was found to hold consensus for several kinases, indicating its potential to affect FUS in vivo [112].

Disruption of the NLS through mutations or PTMs can cause a shift from nuclear to cytoplasmic localization, which promotes the conditions for FUS to pathologically aggregate. If these changes in localization persist, the PTMs may also change as a consequence making it difficult to know cause–effect relationships. Where in the cell modification occurs may be important; phosphorylation of FUS within cytoplasmic RNP bodies may have different consequences than phosphorylation of FUS at sites of DNA damage. Phosphorylation’s connection to disease may also involve molecular mediators, such as transportin-1 or nuclear export machinery.

### 3.2. FUS Methylation

Methylation of lysines and arginines is a common protein modification. It features prominently in the “histone code”—a complex series of modifications that serve as epigenetic regulators of transcription. The RGG domains of FUS contain numerous methylation sites—both theoretical and experimentally identified—which provide potential for very specific and complex regulation of FUS’s activities. Arginine methylation is involved in the nucleocytoplasmic shuttling of many proteins [113], and in recent years has been the focus of much FUS research due to its effects on subcellular localization. It should be noted that, unlike arginine, little experimental evidence supports FUS lysine methylation (Table 1).

Arginine sidechains can exist in several methylation states: unmodified, mono-, or dimethylated. Furthermore, dimethylation can be asymmetric (two methyl groups on a single terminal nitrogen) or symmetric (a methyl group on each terminal nitrogen) [114]. Methylation occurs via protein arginine methyltransferases (PRMTs), which transfer a methyl group from a donor molecule (S-adenosylmethionine) to the guanidino sidechain of arginine. PRMTs are divided into two classes: Type I, which refers to mono- and then dimethylate arginine asymmetrically; and Type II, which refers to mono- and then dimethylate arginine symmetrically. FUS has been shown to be mono- and/or dimethylated by at least two Type I enzymes: PRMT1 and PRMT8 [17,115].

Methylation was first reported by Rappsilber and colleagues in efforts to refine proteomic methods for characterizing arginine methylation. These efforts identified 20 sites of asymmetric dimethylation on FUS [51]. A subsequent mass spectrometry study identified additional arginine asymmetric dimethylation sites, which were proposed to influence FUS’s transcriptional activation activity [17]. These studies found that mono- and dimethylation occur within the three RGG regions (Figure 1). In following studies, a connection between dimethylation and FUS’s subcellular localization was observed, suggesting a link to disease pathogenesis [16,116].

FUS’s C-terminal proline–tyrosine NLS lies at the end of the third RGG region where several arginines govern localization (Figure 1). The NLS and flanking RGG region are recognized by transportin-1. Certain stressors, like hyperosmolar stress or heat shock, cause FUS localization to the cytoplasm as a dimethylated species [16,117]. However, when arginines adjacent to the NLS are demethylated, FUS nuclear localization is restored [117]. Specifically, when mono- or unmethylated, the binding affinity between FUS and transportin-1 is increased resulting in greater nuclear import. This favors nuclear retention of FUS [118], thus arginine methylation regulates the balance between nuclear and cytoplasmic localization.

Both ALS and FTLD-associated FUS pathology appear to be potentiated by the accumulation of FUS in the cytoplasm of affected neurons. Malfunction in transport machinery or disruption of normal methylation can therefore indirectly favor a pathological state. In the case of FUS where the majority of ALS-associated mutations fall within or near the NLS, alteration of the transportin-1 interaction through mutation or through PTMs can affect the ability of FUS to go to the nucleus [117,119]. Case in point, arginine 514 falls within the NLS and is a known site of methylation. For this reason, inhibitors that target PRMTs have been proposed as potential pharmacological agents against FUS-linked disease [120].

Both PRMT1 and PRMT8 co-associate with FUS in cytoplasmic inclusions that contain either wild-type or ALS-associated mutant FUS species [115]. When inclusions are present, a decrease in nuclear PRMT1 concentration occurs, which disrupts normal nuclear functions, including gene repression through histone methylation [121]. Depletion of PRMT1 from the nucleus likely plays a role in the gain-of-function toxicity associated with FUS inclusions. FTLD-FUS and ALS-FUS have different methylation patterns in their disease states [118]. FTLD-FUS cytoplasmic inclusions are primarily mono-methylated and are found to be associated with transportin-1. However, ALS-FUS is dimethylated in inclusions blocking transportin-1 association. This might be due to the presence or absence of FUS mutations in ALS and FTLD, respectively. ALS-causing FUS mutations are frequently in the NLS and increase cytoplasmic localization, whereas mutations are not generally considered to cause FTLD-FUS. However, both mutant and wild-type FUS can be methylated by PRMT1 [115]. 

Methylated FUS can associate into aggregates but is not required for association into stress granules. Because the PrLD and RRM are considered most important for phase separation, it is not clear what the role of arginine methylation is in RNP body formation. An ALS-causing FUS mutation (495X) can form cytoplasmic stress granules regardless of methylation status [122]. However, there is relatively little data examining the role of methylation on FUS liquid–liquid phase separation or aggregation. Given that other domains in PrLD-containing proteins do impact phase separation, the addition of a large number of methyl groups (or at very specific sites) could impact protein self-associations. For example, symmetric dimethylation of the RGG domain in the P-body protein Lsm4 promotes RNP body formation [123]. Interestingly, the pKa of arginine side chains is nearly unchanged after any type of methylation [124]. This suggests that any possible impact of methylation on protein self-association would be due to changes in hydrophobicity or hydrogen-bonding character, not simply bulk changes in electrostatic character.

### 3.3. FUS Ubiquitination and Cleavage

Ubiquitination is used for marking proteins for proteasomal degradation, so it is not surprising that pathological inclusions are frequently ubiquitin-positive. Early neuropathological work with tissue samples from ALS and FTD patients demonstrated the presence of TDP-43-negative, ubiquitin-positive subcellular inclusions in some patient samples [125]. The subtype was called aFTLD-U (atypical frontotemporal lobar degeneration with ubiquitin-positive inclusions). FUS was subsequently identified in these bodies [4] and FUS-positive inclusions have been observed as ubiquitin-positive by several groups [4,126,127,128,129]. These studies show general colocalization of FUS and ubiquitin to these subcellular inclusions, without precisely showing that FUS itself is ubiquitinated in diseased tissue. Because these inclusions are heterogeneous [126], co-localization of ubiquitin and FUS is not sufficient to establish FUS ubiquitination. Early work on FUS suggested that ubiquitination is not a major post-translational modification, but FUS catabolism is regulated by alternative modifications, chiefly phosphorylation [52].

Analysis of FUS in silico, however, does reveal multiple putative ubiquitination sites toward its C-terminus [130]. Additionally, recent work in a motor neuron-like cell model demonstrates that ubiquitination of FUS-positive inclusions temporally follows the formation of early inclusions, consistent with an overall scheme wherein ubiquitin-mediated autophagy regulates clearance of inclusions [126]. Most convincingly, multiple proteomic analyses demonstrate that ubiquitination is detectable in FUS via mass spectrometry, predominately clustered in the RRM domain (Table 1). Extensive polyubiquitination may not be necessary for turnover since the disordered and low-complexity sequences adjacent to the RRM domain could facilitate proteasomal degradation. Overall the data suggest that direct FUS ubiquitination is likely an important PTM. However, the data remain insufficient to definitively establish the precise extent or significance of ubiquitination, especially in relationship to ALS and FTD.

While direct FUS ubiquitination and its role remains unclear [131], other aggregation-prone proteins associated with intracellular inclusions, including TDP-43 [132], are known to be ubiquitinated with important metabolic consequences. Accumulation of cleaved, C-terminal TDP-43 fragments is also observed in pathological inclusions; such accumulation of cleavage products in diseased motor neurons has not been reported for FUS [131,132,133,134,135]. In mass spectrometry studies, FUS N-terminal methionine cleavage and acetylation has been observed in human cell models (Table 1). It remains to be answered how and if cleavage of FUS has any role in pathological aggregation or normal function.

## 4. Future Research into FUS Post-Translational Modification

We have only scratched the surface in understanding the relationship between PTMs and FUS activity and self-association. Experimental evidence suggests that at least 22 different arginines in FUS’s RGG domains can be mono- or dimethylated. Likewise, as many as 32 serines and threonines can be phosphorylated in just the PrLD, not including the additional phosphorylation that occurs in other domains. Therefore, just the PTMs that are experimentally corroborated (not including in silico-predicted sites) suggest that FUS’s biochemical character has a high degree of variability in cells. FUS has numerous functional roles both in and outside of the nucleus, so diverse PTM profiles may support complex specific interactions and functions.

Based on the diverse repertoire of PTMs known to occur, it is likely that additional modifications of FUS have yet to be characterized. The types of PTMs observed with histones, such as arginine deimination and lysine methylation, suggest that additional modifications may occur to FUS. The 20 tyrosines in the repeat motifs of the PrLD are especially prime candidates for modification considering their importance in transcriptional activation and phase separation [110,136]. Identification of tyrosine phosphorylation may be lagging due to technical challenges with these PTMs [137].

Of particular interest is relating different FUS PTMs to different activities and propensity to form solid aggregates. FUS can be recruited into different RNP granules with different functions, but the nuances of how PTMs dictate FUS’s specific inclusion into any given phase-separated structure remains unknown. Additionally, the PTM profile may change depending on subcellular localization or the type of RNP granule to which FUS is recruited. Ultimately, in relationship to disease, the PTMs that are most likely to increase and decrease solid aggregate formation require greater elucidation. Numerous putative PTM sites overlap with sites of disease-associated mutations which could indicate their relevance to disease. However, because we do not know the complete mechanism of how FUS pathology develops, it is a challenge to determine the effects of PTMs. Their precise roles in complex pathological pathways makes it challenging to predict consequences on disease and disentangle cause and effect relationships. To this end, the proximal signals and mechanisms that lead to FUS modification will need to be determined. For example, how DNA damage is relayed to kinases that phosphorylate FUS is unknown. Similarly, the processes that govern FUS methylation through PRMTs require further study.

## Figures and Tables

**Table 1:** Post-translational modifications (PTMs) of FUS.

Amino Acid	Modification	Evidence	Ref.
A2	A	MS^2^	[33,34]
S3	P	MS^2^	[33]
T7	P	MS^1^, MS^2^, NMR	[33,35,36]
T11	P	MS^1^, NMR	[35,36]
T19	*O*-g; P	MS^1^, NMR	[35,36,37]
S26	P	MS^1^, MS^2^, NMR, SEQ, AB	[23,33,36,38]
S30	P	MS^1^, MS^2^, NMR, AB	[33,35,36]
S37	P	MS^2^	[33,36]
S42	P	MS^1^, MS^2^, NMR, SEQ, AB	[23,33,35,36,38]
S54	P	MS^1^	[35]
S57	P	MS^2^	[33]
S61	P	MS^1^, MS^2^, NMR, SEQ	[23,35,36,38]
T68	P	NMR	[36]
T71	P	MS^2^	[36]
S77	P	MS^2^	[33,36]
T78	P	MS^2^	[33,36]
S84	P	MS^1^, NMR, SEQ	[23,35,36,38]
S86	P	MS^2^	[33]
S87	P	MS^1^, MS^2^, NMR	[33,35,36]
S95	P	MS^2^	[33]
S96	P	MS^2^	[33,36]
S108	P	MS^2^	[39]
T109	P	MS^2^	[33,36]
S110	P	MS^2^	[33,36]
S112	P	MS^1^, MS^2^	[33,35]
S115	P	MS^2^	[33]
S117	P	MS^1^, MS^2^, NMR	[33,35,36]
S127	P	MS^2^	[36]
S129	P	MS^2^	[33]
S131	P	MS^1^, MS^2^, SEQ	[23,35,39]
S135	P	MS^2^	[36]
S142	P	MS^1^	[35]
S148	P	MS^2^	[36]
R213	M_1_	MS^2^	[40]
R216	M_1_, M_2_	MS^2^, AB	[17,40,41,42,43,44,45,46,47,48]
R218	M_1_, M_2_	MS^2^, AB	[17,40,41,42,43,44,45,46,47,48]
S221	P	MS^2^	[39,43,49,50]
Y232	P	MS^2^	[43]
R234	M_1_	MS^2^	[41]
R242	M_1_, M_2_	MS^2^	[17,40,41,42,43,51]
R244	M_1_, M_2_	MS^2^	[41,42,51]
R248	M_1_, M_2_	MS^2^	[41,42,51]
R251	M_2_	MS^2^	[42,51]
S256*	P	MUT	[52]
R259	M_1_, M_2_	MS^2^	[40,41,42,43,51]
K264	U	MS^2^	[53]
R269	M_1_	MS^2^	[41]
S273	P	MS^2^	[43]
S277	P	MS^2^	[43,54,55,56,57,58,59]
T286	P	MS^2^	[58,59,60]
Y304	P	BP^1^, BP^2^	[61,62]
K316	U	MS^2^	[43,63,64,65]
T317	P	MS^2^	[66]
Y325	P	MS^2^, BP^1^, BP^2^	[43,61,62,66,67]
T326	P	MS^2^	[66,67]
K332	A	MS^2^	[53]
K334	U	MS^2^	[53]
S340	P	MS^2^	[49,54,56,68,69]
S346	P	MS^2^	[54,56,58,67]
K348	U	MS^2^	[64]
K357	A; U	MS^2^	[43,53]
S360	P	MS^2^	[54]
K365	M_1_; U	MS^2^	[63,65,70]
R371	M_1_	MS^2^	[40,41,42]
R377	M_2_	MS^2^	[51]
R383	M_1_, M_2_	MS^2^	[41,51]
R386	M_2_	MS^2^	[51]
R388	M_2_	MS^2^	[51]
R394	M_1_, M_2_	MS^2^	[17,40,41,43,44,51]
Y397	P	MS^2^	[71]
R407	M_1_, M_2_	MS^2^	[40,41,43,51]
S439	P	MS^2^	[54]
K448	U	MS^2^	[53]
S462	P	MS^2^	[43,53,54,72,73]
Y468	P	MS^2^	[43,67,74,75]
R472	M_1_	MS^2^	[44]
R473	M_1_, M_2_	MS^2^	[40,44,51]
R476	M_1_, M_2_	MS^2^	[41,44,51]
R481	M_1_, M_2_	MS^2^	[41,43,51]
R485	M_1_, M_2_	MS^2^	[41,43,51]
R487	M_1_, M_2_	MS^2^	[41,51]
R491	M_2_	MS^2^	[46,51]
R495	M_1_, M_2_	MS^2^	[40,41,46,51]
R498	M_2_	MS^2^	[46,51]
R503	M_1_, M_2_	MS^2^	[40,41,42,43,44,46,51]
R514	M_1_	MS^2^	[40,41,42]
Y526	P	AB	[76]

Acetylation (A); GalNAc O-glycosylation (*O*-g); Monomethylation (M_1_); Dimethylation (M_2_); Phosphorylation (P); Ubiquitination (U). Mass spectrometry of recombinant protein (MS^1^); mass spectrometry of cellular protein (MS^2^); NMR of recombinant protein (NMR); protein sequencing (SEQ); bioinformatic prediction using Phospho Motif Finder (BP^1^); bioinformatic prediction using NetPhos 3.1 (BP^2^); *FUS isoform 2; antibody specific to PTM (AB); site-specific mutations (MUT); site of post translational modification and an ALS-associated mutation (grey background). Many additional sites are predicted in silico, but lack experimental corroboration.

## Abbreviations

ALS	Amyotrophic lateral sclerosis
FTD	Frontotemporal dementia
FTLD	Frontotemperal lobar degeneration
FUS	Fused in sarcoma
LLPS	Liquid–liquid phase separation
PrLD	Prion-like domain
PTM	Post-translational modification
RRM	RNA recognition motif
